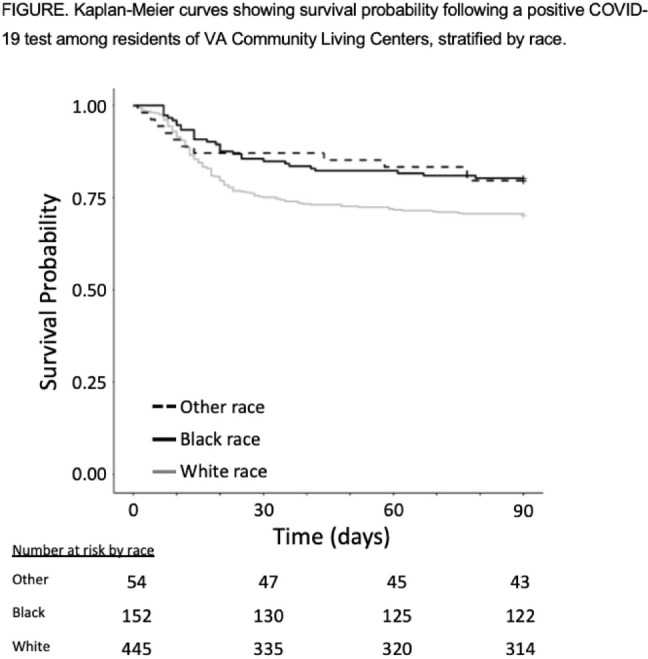# Absence of racial and ethnic disparities in COVID-19 survival among residents of US Veterans’ Affairs community living centers

**DOI:** 10.1017/ash.2022.121

**Published:** 2022-05-16

**Authors:** Mayyadah Alabdely, Sonya Kothadia, Taissa Bej, Brigid Wilson, Sunah Song, Ukwen Akpoji, Federico Perez, Robin Jump

## Abstract

**Background:** COVID-19 has had a disproportionate effect on nursing homes residents as well as people from racial and ethnic minorities. Whether differences in mortality due to COVID-19 exists for nursing-home residents from racial and ethnic minorities is less clear, with some previous studies reporting systems-level disparities. The Department of Veterans’ Affairs (VA) has nursing homes, termed community living centers (CLCs), across the United States. We hypothesized that differences in COVID-19–related mortality among racial and ethnic minorities would be less pronounced among residents of CLCs. **Methods:** Using data from the VA Corporate Data Warehouse, we conducted a retrospective cohort study from April 14, 2020 (implementation of population-based testing) to December 10, 2020 (availability of a COVID-19 vaccine). Inclusion criteria were residents with a positive SARS-CoV-2 test while residing in or <48 hours before admission to a CLC. Positive tests <180 days after a prior positive test were excluded. We assessed the cohort for demographics, including self-reported race or ethnicity, clinical characteristics, and survival probability including all-cause 30-day mortality. A multivariable logistic regression model was used to estimate odds ratios (ORs) and 95% confidence intervals (CIs) for all-cause 30-day mortality that included race, ethnicity, age, and Charlson comorbidity index (CCI). **Results:** Among 14,759 CLC residents, 651 (4.4%) had a positive SARS-COV-2 test. Their mean age was 75.7 ± 11.3 years, and self-reported race or ethnicity was 68% White (445 of 651), 23% Black (152 of 651), and 4% Hispanic/Latinx (27 of 651). The mean CCI was lower among White residents than Black residents (4.15 ± 2.6 vs 4.61 ± 3.1, respectively). All-cause 30-day mortality for CLC residents following positive SARS-COV-2 test was 25% for White patients, 14% for Black patients, and 15% for Hispanic/Latinx patients (Fig. 1). Age (in years), but neither race or ethnicity nor CCI, was independently associated with all-cause 30-day mortality (OR, 1.07; 95% CI, 1.05–1.09) in CLC residents with COVID-19. **Conclusions:** Among VA CLC residents with a positive COVID-19 test, minority CLC residents did not have worse outcomes than white residents, suggesting that users of the VA healthcare system may enjoy abrogation of some aspects of health disparities.

**Funding:** None

**Disclosures:** None

**Figure:**